# Dual-5α-Reductase Inhibition Promotes Hepatic Lipid Accumulation in Man

**DOI:** 10.1210/jc.2015-2928

**Published:** 2015-11-17

**Authors:** Jonathan M. Hazlehurst, Andrei I. Oprescu, Nikolaos Nikolaou, Riccardo Di Guida, Annabel E. K. Grinbergs, Nigel P. Davies, Robert B. Flintham, Matthew J. Armstrong, Angela E. Taylor, Beverly A. Hughes, Jinglei Yu, Leanne Hodson, Warwick B. Dunn, Jeremy W. Tomlinson

**Affiliations:** Oxford Centre for Diabetes, Endocrinology, and Metabolism (J.M.H., N.N., L.H., J.W.T.), National Institute for Health Research Oxford Biomedical Research Centre, University of Oxford, Churchill Hospital, Oxford OX3 7LE, United Kingdom; Centre for Diabetes, Endocrinology, and Metabolism (A.I.O., A.E.T., B.A.H.), Institute of Biomedical Research, School of Clinical and Experimental Medicine, School of Biosciences and Regional Phenome Centre (R.D.G., W.B.D.), Centre for Liver Research and National Institute for Health Research Liver Biomedical Research Unit (M.J.A.), and School of Sports and Exercise Sciences (J.Y.), University of Birmingham, Birmingham B15 2TH, United Kingdom; National Institute for Health Research/Wellcome Trust Clinical Research Facility (A.E.K.G.), Queen Elizabeth Hospital, Birmingham B15 2TT, United Kingdom; and Department of Medical Physics (N.P.D., R.B.F.), Queen Elizabeth Hospital, Birmingham B15 2GW, United Kingdom

## Abstract

**Context:**

5α-Reductase 1 and 2 (SRD5A1, SRD5A2) inactivate cortisol to 5α-dihydrocortisol in addition to their role in the generation of DHT. Dutasteride (dual SRD5A1 and SRD5A2 inhibitor) and finasteride (selective SRD5A2 inhibitor) are commonly prescribed, but their potential metabolic effects have only recently been identified.

**Objective:**

Our objective was to provide a detailed assessment of the metabolic effects of SRD5A inhibition and in particular the impact on hepatic lipid metabolism.

**Design:**

We conducted a randomized study in 12 healthy male volunteers with detailed metabolic phenotyping performed before and after a 3-week treatment with finasteride (5 mg od) or dutasteride (0.5 mg od). Hepatic magnetic resonance spectroscopy (MRS) and two-step hyperinsulinemic euglycemic clamps incorporating stable isotopes with concomitant adipose tissue microdialysis were used to evaluate carbohydrate and lipid flux. Analysis of the serum metabolome was performed using ultra-HPLC-mass spectrometry.

**Setting:**

The study was performed in the Wellcome Trust Clinical Research Facility, Queen Elizabeth Hospital, Birmingham, United Kingdom.

**Main Outcome Measure:**

Incorporation of hepatic lipid was measured with MRS.

**Results:**

Dutasteride, not finasteride, increased hepatic insulin resistance. Intrahepatic lipid increased on MRS after dutasteride treatment and was associated with increased rates of de novo lipogenesis. Adipose tissue lipid mobilization was decreased by dutasteride. Analysis of the serum metabolome demonstrated that in the fasted state, dutasteride had a significant effect on lipid metabolism.

**Conclusions:**

Dual-SRD5A inhibition with dutasteride is associated with increased intrahepatic lipid accumulation.

The worldwide burden of metabolic disease, including its hepatic manifestation, nonalcoholic fatty liver disease (NAFLD), is escalating. NAFLD is associated with significant morbidity and mortality (1), and there is an unmet clinical need to understand its pathogenesis to improve treatment. Both glucocorticoids (GCs) and androgens have been implicated in its pathogenesis; circulating GC excess (Cushing's syndrome) and T deficiency in men and excess in women are associated with NAFLD (2–5).

Tissue-specific actions of GCs and androgens (notably in liver and adipose) are controlled at a prereceptor level by a series of enzymes that regulate the availability of steroid hormones to bind and activate their cognate receptors. The A-ring reductases include 5α-reductase type 1 [SRD5A1], type 2 [SRD5A2], and 5β-reductase. SRD5A1 and SRD5A2 reduce δ^4,5^,3-oxosteroid hormones to their 5α-reduced metabolites (6) and are critical in this process. They inactivate cortisol to 5α-dihydrocortisol, which is subsequently converted in a nonrate-limiting step to 5α-tetrahydrocortisol by 3α-hydroxysteroid dehydrogenase. Additionally, SRD5A1 and SRD5A2 activate T to the more potent androgen, DHT. Both SRD5A1 and SRD5A2 are expressed in human hepatocytes (7), and SRD5A1 alone is expressed in adipocytes (8).

The precise contribution of each isoform to GC metabolism is not clearly defined. Both are able to metabolize cortisol (but not cortisone). Patients with mutations in SRD5A2 present with 46XY disorder of sexual differentiation and have been shown to have markedly reduced 5α-reduced GC metabolites (9), although the metabolic phenotype in these patients has not been examined. Currently patients with functional mutations in SRD5A1 that compromise enzyme activity have not been identified.

Several cross-sectional and interventional studies have highlighted a link between 5α-reductase and metabolic phenotype. Enhanced 5α-reductase activity is seen in patients with obesity (10) and polycystic ovary syndrome (11) correlating with measures of insulin resistance (12). We have shown that 5α-reductase activity is increased in patients with biopsy-proven hepatic steatosis but not in those with cirrhosis and have proposed that increased 5α-reductase activity may act as a protective mechanism preventing progression of metabolic phenotype within the liver through increased local clearance of GCs (13). Whereas these studies have been important in highlighting the relationship between 5α-reductase and metabolic phenotype, they have been unable to resolve whether these observations reflect cause or consequence of disease. Rodent studies have begun to help answer these questions and suggest that selective deletion of SRD5A1 may be detrimental (14, 15)

This issue is clinically relevant, not only due to the prevalence of NAFLD and the importance of understanding its pathogenesis but also with the widespread use of 5α-reductase inhibitors in clinical practice. The dual SRD5A1 and SRD5A2 inhibitor, dutasteride, and the selective SRD5A2 inhibitor, finasteride, are extensively used in the treatment of benign prostatic hyperplasia, whereby they decrease local generation of DHT. To date, only a single study has extensively examined the potential metabolic impact of 5α-reductase inhibition, and although a detrimental impact on insulin sensitivity was observed, the impact on the liver remains unclear (16).

We have undertaken a detailed metabolic study to determine the potential effects of 5α-reductase inhibition upon the metabolic phenotype, specifically its potential to regulate lipid metabolism within the liver and to identify the mechanisms that may underpin these observations. In our exploratory study, we used paired hepatic magnetic resonance, hyperinsulinaemic-euglycaemic clamps incorporating stable isotopes, adipose microdialysis, and an analysis of the serum metabolome to examine the effects of SRD5A inhibition in detail.

## Patients and Methods

### Clinical protocol

The clinical protocol received full ethical approval from the South Birmingham Local Research Ethics Committee (reference 12/WM/0122). Twelve healthy male volunteers (mean age 36.3 ± 4.4 y, body mass index [BMI] 26.6 ± 1.2 kg/m^2^) were recruited from local advertisement and provided written informed consent. All were nondiabetic, had not been on GC therapy within the previous 6 months, were normotensive, were not on any drugs known to impact upon GC metabolism, were aged 18–65 years, and had a BMI between 20 and 35 kg/m^2^. The clinical protocol is summarized below. The decision to recruit 12 healthy volunteers (six per group) was based on a priori power calculations (for 80% power) extrapolated from published rodent data (14) that identified a 49.2% increase in hepatic lipid content in SRD5A1 knockout mice. Although there are limitations in extrapolating rodent data into the clinical setting, calculations suggested that at least five volunteers in each group would be required.

At 5:00 pm, volunteers attended the Wellcome Trust Clinical Research Facility (Birmingham, United Kingdom), having provided a 24-hour urinary collection. Total body water was estimated using bioimpedance (model BC418MA; Tanita), and volunteers were given oral ^2^H_2_O (3 g/kg total body water in two divided doses 6:00 and 10:00 pm) followed by drinking water enriched to 0.4% to determine the rates of hepatic de novo lipogenesis (DNL). A standardized meal was provided at 6:00 pm (carbohydrate 45 g, protein 23 g, and fat 20 g), and after this point volunteers were fasted until completion of the hyperinsulinemic clamp the following day. The following morning a 3T-magnetic resonance spectroscopy (MRS) of the liver was performed to quantify hepatic lipid content (see below). At 9:00 am, an adipose microdialysis catheter (CMA Microdialysis) was inserted under local anesthetic 10 cm lateral to the umbilicus, and samples (0.3 μL/min) were taken every 30 minutes until completion of the hyperinsulinemic clamp.

On commencement of the two-step hyperinsulinemic euglycemic clamp, a bolus of U-^13^C-glucose (CK Gas Ltd) was administered (2 mg/kg) over 1 minute followed by a constant infusion (0.02 mg/kg · min). Concurrently, an infusion of ^2^H_5_-glycerol was started (0.1 μmol/kg · min) to allow assessment of whole-body lipolytic rates (rate of endogenous glycerol appearance [Ra glycerol ]). The basal phase continued for 2 hours prior to commencement of insulin and glucose infusions. Basal steady-state samples were taken at three time points during the last 30 minutes of the basal phase. At 2 hours, insulin (Actrapid; Novo Nordisk) was infused at 20 mU/m^2^ · min alongside an infusion of 20% glucose enriched with U-^13^C-glucose to 4%. This low-dose insulin phase continued for 2 hours. For the remainder of the hyperinsulinemic euglycemic clamp, arterialized blood samples to measure glucose were taken at 5-minute intervals, and the glucose infusion rate was adjusted to maintain fasting glycemic levels. Steady-state samples were taken at three time points in the final 30 minutes of this low-dose insulin phase. At the completion of this phase, the insulin infusion rate was increased to 100 mU/m^2^ · min for the following 2 hours. Steady-state samples were obtained in this high-dose insulin phase as described above. Rates of glucose disposal were calculated using a modified version of the Steele equations (17, 18). Volunteers were then randomized, but not blinded, to receive treatment with either finasteride 5 mg or dutasteride 0.5 mg daily for 3 weeks after which time all investigations were repeated. Randomization was performed by statisticians who were not part of the investigative team. Although the primary investigators were not blinded to the treatments, the analysis of key areas including MRS, stable isotopes, and serum metabolome was performed by researchers blinded to the specific treatment.

### Biochemical and stable isotope analysis

Liver biochemistry, electrolytes, urea, creatinine, cholesterol, triglycerides, and full blood counts were measured using standard laboratory methods (Roche modular system; Roche Ltd). Cortisol (R&D Systems), insulin (Mercodia), and nonesterified fatty acids (Zen-Bio) were measured using commercially available assays according to the manufacturers' instructions.

Microdialysate samples were collected in microvials and analyzed using a mobile photometric, enzyme-kinetic analyzer (CMA Iscus Flex) for glucose, pyruvate, lactate. and glycerol.

The enrichment of U-^13^C-glucose and D_5_-glycerol (1,1,2,3,3-D_5_) in plasma was determined by gas chromatography-mass spectrometry (model 5973; Agilent Technologies). Deuterium enrichment of the total body water pool was measured using the Gasbench II coupled online to a ThermoFinnigan Deltaplus XP isotope ratio mass spectrometer and the palmitate fraction of total plasma triglycerides using an automated gas chromatography high temperature conversion isotope ratio mass spectrometer ThermoFinnigan Delta Pus XP (ThermoFinnigan) (19).

### Calculation of the contribution of de novo synthesized palmitate to plasma triglyceride

The proportion of endogenous palmitate synthesized de novo was calculated from the incorporation of ^2^H (from ^2^H_2_O) in the palmitate present in the plasma triglyceride pool, and this was calculated using the following formula: fraction = (Δ^2^H to ^1^H ratio in palmitate methyl ester/Δ^2^H to ^1^H ratio in water pool) (34/22), where 34 is the total number of H atoms in palmitate methyl ester and 22 is the number of water molecules incorporated into palmitate via DNL (20–22).

### MRS determination of hepatic lipid fraction

The method to determine intrahepatic lipid with MRS is included in the Supplemental Methods.

### Serum and urine steroid metabolite analysis

Serum steroids were measured by liquid chromatography/mass spectrometry. Briefly, steroids were extracted from serum (after the addition of the internal standard) via liquid/liquid extraction using tert-methyl butyl ether as described previously (23). However, to ensure T and DHT could be accurately quantified above the lower limit of quantification (LLOQ), the volume used for steroid extraction was increased to 2000 μL, which was reconstituted in 125 μL before analysis. The tert-methyl butyl ether layer was removed, evaporated, and reconstituted in 50:50 methanol/water prior to liquid chromatography/mass spectrometry analysis. Steroids were quantified relative to a calibration series via tandem mass spectrometry. The calibrators and quality controls are prepared in-house from steroids (Sigma-Aldrich) and tested against reference standards (ChromSystems). A Waters Xevo mass spectrometer with an electrospray ionization source was used with an attached Acquity liquid chromatography system. Steroids were eluted from a HSS T_3_, 1.8-μm, 1.2- × 50-mm column using a methanol/water gradient system with 0.1% formic acid. The LLOQs for individual steroids were cortisol 0.7 nmol/L, cortisone 0.7 nmol/L, T 2 nmol/L, and DHT 0.25 nmol/L. The coefficient of variation for all assays was less than 20% (based on more than six replicate extractions). The LLOQs quoted above are based on nonconcentrated samples.

Urinary steroids were analyzed by gas chromatography-mass spectrometry. A detailed description of this methodology has been published previously (24).

### Serum metabolic profiling

Details of the method for the serum metabolic profiling are included in the Supplemental Data.

### Statistical approach

Data are presented as mean ± SE unless otherwise stated. Sample size calculations were based on the primary end point of change in hepatic lipid content. Additional secondary outcomes included change in hepatic and peripheral insulin sensitivity and adipose tissue lipid mobilization. Area under the curve (AUC) analysis was performed using the trapezoidal method. Paired *t* tests were used (or nonparametric equivalents when appropriate) to compare individual variables (eg, MRS and DNL) between before and after intervention within each patients group. When variables were compared between independent groups, ie, substituting dutasteride for finasteride in one of the two groups nonpaired tests were used. For analysis of repeated samples (eg, changes in adipose microdialysis) (either during an individual investigation or for comparison of the same investigation between baseline and after 3 wk of the intervention), a two-way, repeated-measures ANOVA was used with Bonferroni's correction for multiple analyses. All analyses were performed using the GraphPad statistical software.

## Results

### Whole-body insulin sensitivity is unchanged by treatment with dutasteride or finasteride

**Figure 1.**
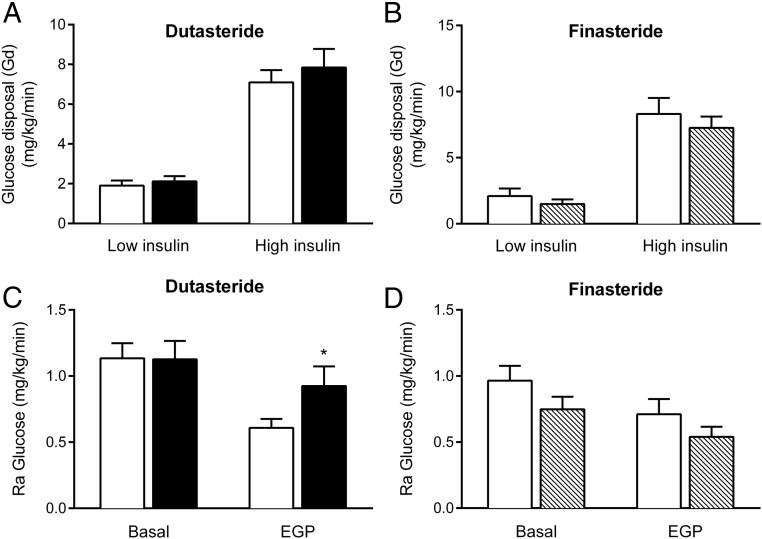
The effect of 5α-reductase inhibition on glucose disposal and glucose production. Data shown are dutasteride (A and C) and finasteride (B and D) on Gd (A and B) and Ra glucose (C and D). Open bars, Pretreatment and filled bars, the effect of 3 weeks of drug treatment; black, dutasteride; shaded, finasteride. *, *P* < .05 vs pretreatment.

Over the 3 weeks of the study, dutasteride and finasteride treatment had no effect on anthropometric measurements (Table 1). Dutasteride, but not finasteride, decreased systolic blood pressure (BP) (dutasteride: 143 ± 8.2 mm Hg vs 128 ± 3.6 mm Hg, *P* < .05; finasteride: 144 ± 8 mm Hg vs 141 ± 7.8 mm Hg, *P* = .8) with no effect on diastolic BP or electrolytes. There was no effect on fasting glucose or insulin levels (Table 1). During the hyperinsulinemic euglycemic clamp, glucose infusion rates, either unadjusted or corrected for circulating insulin levels, were not different before or after dutasteride or finasteride treatment (Table 2). In addition, glucose disposal (Gd), largely reflecting skeletal muscle insulin sensitivity, did not change in either treatment arm (Table 2 and Figure 1, A and B).

**Table 1.** Demographics, Anthropometric Data, and Fasting Biochemistry in Male Volunteers Before and After Randomization to 21 Days of Treatment With Either Finasteride (5 mg once a day) or Dutasteride (0.5 mg once a day)

Clinical Variable	Finasteride		Dutasteride	
	Before	After	Before	After
Age, y	36.7 ± 8.2	36.7 ± 8.2	36.0 ± 4.3	36.0 ± 4.3
Weight, kg	98.6 ± 7.5^a^	98.6 ± 7.4^a^	76.2 ± 4.4^a^	76.0 ± 4.0^a^
BMI, kg/m^2^	29.6 ± 1.8	29.8 ± 1.8	24.2 ± 1.9	24.1 ± 0.7
Total fat, %	22.6 ± 2.6	23.2 ± 2.7	16.1 ± 1.5	16.5 ± 1.9
Total fat mass, kg	22.9 ± 4.4^a^	23.5 ± 4.3^a^	12.4 ± 1.5^a^	12.5 ± 1.5^a^
Total fat-free mass, kg	75.7 ± 4.5	75.2 ± 4.5	63.9 ± 3.5	63.5 ± 3.6
SBP, mm Hg	143.5 ± 8.0	140.8 ± 7.8	143.0 ± 8.2^b^	128.0 ± 3.6^b^
DBP, mm Hg	87.5 ± 3.5^a^	84.3 ± 6.9	78.2 ± 2.4^a^	71.5 ± 5.0
Glucose, mmol/L	4.4 ± 0.2	4.5 ± 0.2	4.4 ± 0.1	4.5 ± 0.1
Insulin, pmol/L	27.1 ± 7.5	29.3 ± 8.6	14.6 ± 4.5	16.7 ± 3.9
HDL cholesterol, mmol/L	1.5 ± 0.1	1.5 ± 0.1	1.4 ± 0.1	1.2 ± 0.2
Total cholesterol, mmol/L	5.5 ± 0.5	5.0 ± 0.7	5.5 ± 0.8	5.5 ± 0.9
Triglycerides, mmol/L	1.1 ± 0.1	0.9 ± 0.1	1.0 ± 0.1	1.5 ± 0.3
AST, U/L (5–43)	21.0 ± 2.0	21.2 ± 2.4	24.7 ± 3.5	23.5 ± 4.1
Bilirubin, mol/L (<22)	10.3 ± 1.9	10.4 ± 1.3	17.0 ± 4.2	17.4 ± 5.5
Hemoglobin, g/dL (13.5–18.0)	14.6 ± 0.3^b^	13.8 ± 0.4^b^	14.8 ± 0.3^b^	13.9 ± 0.3^b^
Cortisol, nmol/L (55–580)	334 ± 37^b^	196 ± 25^a,b^	293 ± 26	276 ± 19^a^
Cortisone, nmol/L (17–97)	78.7 ± 4.3	62.2 ± 4.4^a^	81.1 ± 4.3	76.9 ± 4.6^a^
Androstenedione, nmol/L (1.7–7.7)	2.6 ± 0.4	2.9 ± 0.5	2.8 ± 0.4	3.8 ± 0.3
T, nmol/L (10–35)	17.7 ± 2.7	20.4 ± 2.4	14.6 ± 1.0^b^	22.3 ± 2.1^b^
DHT, nmol/L (0.3–2.4)	0.9 ± 0.2^c^	0.2 ± 0.1^c^	0.7 ± 0.2	0.2 ± 0.0.1

Abbreviations: AST, aspartate aminotransferase; DBP, diastolic BP; HDL, high-density lipoprotein; SBP, systolic BP. Local references are included in parentheses.

a *P* < 0.05, dutasteride vs finasteride.

b *P* < .05, before vs after treatment.

c *P* < .01, before vs after treatment.

**Table 2.** The Effect of 5α-Reductase Inhibition on Glucose and Lipid Metabolism During a Two-Step Hyperinsulinemic Euglycemic Clamp

Metabolic Variable	Finasteride		Dutasteride	
	Before	After	Before	After
NEFAs, μmol/L				
Basal	1117 ± 66	1118 ± 53	1190 ± 72	1129 ± 82
Low insulin	708 ± 44	674 ± 13	681 ± 17	679 ± 14
High insulin	654 ± 15	639 ± 7	638 ± 5	658 ± 14
M value, mg/kg · min				
Low insulin	3.74 ± 0.77	3.16 ± 0.52	3.23 ± 0.36	3.38 ± 0.51
High insulin	11.11 ± 1.18	10.27 ± 0.69	9.87 ± 0.66	10.23 ± 1.06
Mfi value, mg/kg · min · pmol · L				
Low insulin	0.028 ± 0.007	0.027 ± 0.009	0.038 ± 0.012	0.035 ± 0.009
High insulin	0.011 ± 0.002	0.012 ± 0.002	0.015 ± 0.003	0.015 ± 0.003
Ra glucose, mg/kg · min				
Basal	0.923 ± 0.125	0.748 ± 0.095	1.134 ± 0.114	1.127 ± 0.139
EGP, mg/kg · min				
Low insulin	0.711 ± 0.12	0.540 ± 0.08	0.609 ± 0.07^a^	0.924 ± 0.15^a^
Gd, mg/kg · min				
Low insulin	2.09 ± 0.58	1.49 ± 0.35	1.90 ± 0.26	2.02 ± 0.40
High insulin	8.30 ± 1.22	7.25 ± 0.85	7.09 ± 0.62	7.84 ± 0.93
Ra glycerol, mg/kg · min				
Basal	0.28 ± 0.06	0.21 ± 0.02	0.25 ± 0.02	0.27 ± 0.08
Low insulin	0.20 ± 0.09	0.11 ± 0.03	0.07 ± 0.02	0.07 ± 0.02
High insulin	0.18 ± 0.08	0.13 ± 0.04	0.06 ± 0.01	0.08 ± 0.02
DNL, fractional incorporation of ^2^H_2_O into palmitate, %				
Basal	1.6 ± 0.5	1.3 ± 0.3	1.2 ± 0.3	6.5 ± 2.7
Adipose microdialysis				
Glycerol AUC, μmol/L · h				
Basal	224 ± 47	259 ± 16	266 ± 24	220 ± 40
Low insulin	151 ± 37	161 ± 22	181 ± 31^a^	105 ± 22^a^
High insulin	94 ± 28	86 ± 15	99 ± 19	64 ± 16
Pyruvate AUC, μmol/L · h				
Basal	54.4 ± 14	57.7 ± 13	61.4 ± 21	37 ± 10
Low insulin	104 ± 19	110 ± 17	134 ± 30	71 ± 11
High insulin	105 ± 9	131 ± 18	141 ± 27	92 ± 16
Lactate AUC, μmol/liter · h				
Basal	0.96 ± 0.2	0.92 ± 0.1	0.9 ± 0.2	1.3 ± 0.3
Low insulin	1.94 ± 0.3	1.76 ± 0.2	2.2 ± 0.3	1.8 ± 0.3
High insulin	2.67 ± 0.5	2.64 ± 0.3	2.9 ± 0.4	2.2 ± 0.4
Glucose AUC, mmol/liter · h				
Basal	3.3 ± 0.6	3.6 ± 0.4	4.4 ± 0.4	4.6 ± 0.5
Low insulin	4.0 ± 0.6	4.0 ± 0.4	4.0 ± 0.5	4.5 ± 0.3
High insulin	4.1 ± 0.7	4.5 ± 0.5	3.3 ± 0.6	5.0 ± 0.4

Abbreviations: EGP, endogenous glucose production. Data are before and after randomization to 21 days of treatment with either finasteride (5 mg once a day) or dutasteride (0.5 mg once a day).

a *P* < .05, before vs after treatment.

### Hepatic insulin sensitivity and lipid accumulation

Hepatic glucose production rate at baseline was not affected by either dutasteride or finasteride (Table 2 and Figure 1, C and D). The endogenous glucose production rate was significantly increased after dutasteride only (dutasteride before vs after: 0.61 ± 0.07 mg/kg · min vs 0.92 ± 0.15 mg/kg · min, *P* = .046; finasteride: 0.71 ± 0.12 vs 0.54 ± 0.08, *P* = .18), consistent with increased hepatic insulin resistance (Figure 1, C and D).

**Figure 2.**
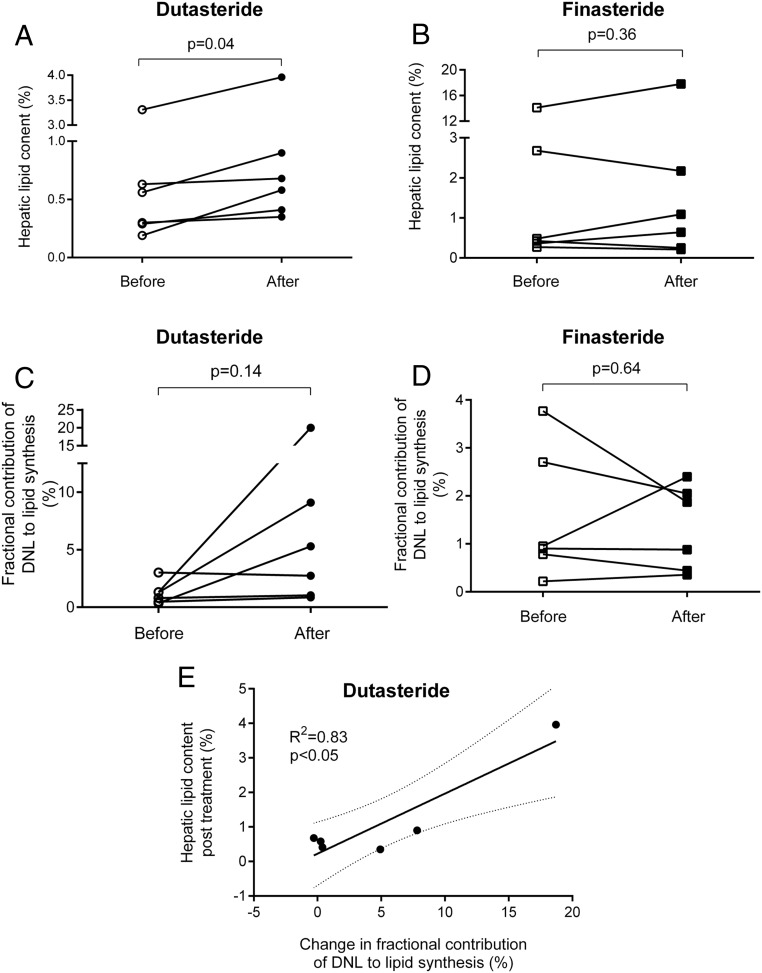
The impact of 5α-reductase inhibition on de novo lipogenesis and hepatic lipid content. Data shown are the effect on dutasteride (A and C) and finasteride (B and D) on hepatic lipid content percentage as measured by MRS (A and B) and DNL as measured by deuterated water incorporation into plasma triglyceride palmitate (C and D). Open circles/squares represent pretreatment and filled circles/squares represent the effect of 3 weeks of drug treatment (circles, dutasteride; squares, finasteride). *, *P* < .05 vs pretreatment. The change in rate of DNL is positively correlated with final liver lipid content after treatment with dutasteride (E) but not finasteride.

The proton density fat fraction (single echo time) increased in all volunteers treated with dutasteride, whereas in those patients treated with finasteride, only three of the six had an increase in proton density fat fraction (percentage lipid content) (dutasteride before vs after: 0.88 ± 0.35 vs 1.14 ± 0.40, *P* = .041; finasteride: 3.05 ± 1.58 vs 3.69 ± 2.0, *P* = .35) (Figure 2, A and B). When corrected for T_2_ decay, borderline significance was achieved (percentage lipid content, dutasteride: 0.58% ± 0.21% vs 0.75% ± 0.24%, *P* = .056; finasteride: 2.39% ± 1.25% vs 3.08% ± 1.72%, *P* = .37).

The relative (percentage) contribution of de novo synthesized palmitate to plasma triglycerides increased in five of six participants treated with dutasteride and in only two of six of those randomized to finasteride, but this did not reach significance (DNL percentage dutasteride before vs after: 1.21% ± 0.4% vs. 6.51% ± 2.7%, *P* = .14; finasteride: 1.55% ± 0.5% vs 1.33% ± 0.3%, *P* = .65 (Figure 2, C and D, and Table 2). However, in those patients treated with dutasteride (and not finasteride), the change in percentage DNL correlated positively with the change in hepatic lipid content as measured by MRS (dutasteride: R^2^ = 0.84, *P* = .0109; finasteride: R^2^ = 0.06, *P* = .6) (Figure 2E).

### Adipose tissue function

**Figure 3.**
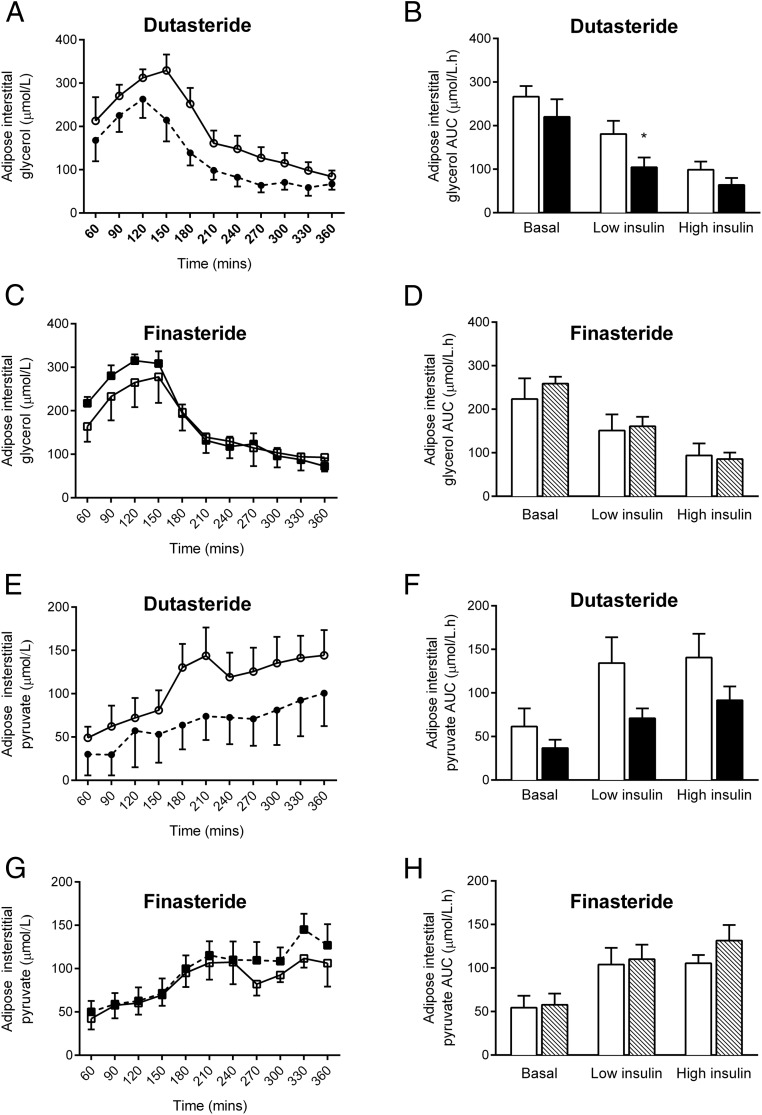
The effect of 5α-reductase inhibition on sc abdominal adipose tissue. Dutasteride enhances insulin-mediated suppression of lipolysis in abdominal sc adipose tissue as measured by adipose interstitial fluid release of glycerol into the microdialysate (A and B). In contrast, finasteride is without effect (C and D). Similarly, dutasteride decreases insulin-stimulated pyruvate release into adipose interstitial fluid (E and F), whereas finasteride is without effect (G and H). Data are presented across the duration of the hyperinsulinemic euglycemic clamp (A, C, E, and G) and also expressed as AUC for the basal, low insulin, and high insulin phases (B, D, F, and H). Open circles/squares/bars represent pretreatment and filled circles/squares/bars represent the effect of 3 weeks of drug treatment (circles or black bars, dutasteride; squares or shaded bars, finasteride). *, *P* < .05 vs pretreatment.

Neither dutasteride nor finasteride altered insulin-mediated suppression of circulating nonesterified fatty acids (NEFAs) (Table 2). In addition, Ra glycerol, as a measure of systemic lipolysis, was not changed (Table 2). However, release of glycerol into sc adipose tissue interstitial fluid was reduced by dutasteride and not finasteride after the low-dose insulin infusion (Figure 3 and Table 2). There was no significant effect of the treatment of interstitial pyruvate, glucose, or lactate (Table 2). There was a trend toward decreased interstitial pyruvate release with treatment with dutasteride, which did not reach significance (Figure 3 and Table 2).

### Serum metabolic phenotyping

**Figure 4.**
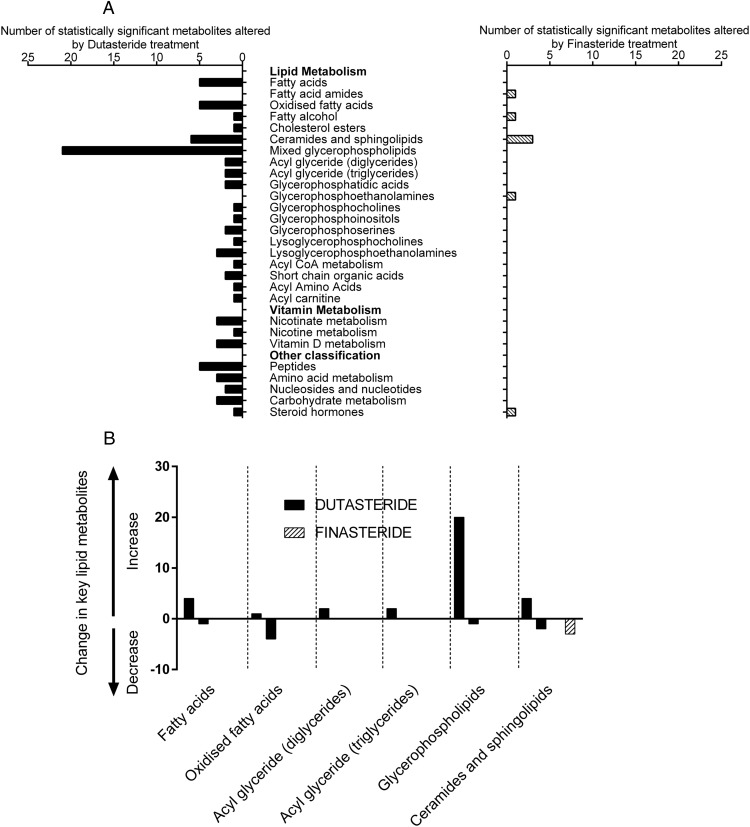
Serum metabolome analysis in healthy volunteers treated with either dutasteride or finasteride. The total number of metabolites differentially regulated in the fasting state before and after 3 weeks of a drug intervention is significantly greater in those patients treated with dutasteride, with a predominant effect on lipid metabolites (A). Classes of lipid metabolites are differentially regulated (either up- or down-regulated) by dutasteride and finasteride (B).

Dutasteride and not finasteride markedly changed the serum metabolome in the fasted state. In this nontargeted whole-metabolome approach, results are expressed as changes of different classes of metabolites rather than individual metabolites. Dutasteride treatment was associated with a significant increase in 98 serum metabolites and a reduction in 25 metabolites, whereas finasteride had a more modest effect (six increased; five decreased) (Figure 4A). The classes of metabolites changed by dutasteride were mainly lipids with particular increases in glycerophospholipids (Figure 4B).

Both dutasteride and finasteride differentially regulated the response of the serum metabolome to insulin. In the finasteride arm, prior to treatment, 249 metabolites were significantly regulated by insulin, and subsequently only 132 of these were regulated by insulin after treatment with finasteride (*P* < .05). In the dutasteride arm, 269 unique metabolites were regulated by insulin prior to treatment. Two hundred sixteen metabolites continued to be regulated by insulin after treatment with dutasteride (*P* < .06) (Supplemental Figure 1).

### Steroid metabolite analysis

Complete 24-hour urinary steroid profile analysis is presented in Supplemental Table 1. The 5α tetrahydrocortisol ratio is an appropriate proxy of SRD5A activity and does not differ between groups at baseline. As expected, dutasteride and finasteride both resulted in a significant reduction in 5α-reduced steroids. Consistent with decreased adrenal output as a consequence of reduced cortisol clearance, total cortisol metabolites were reduced by both treatments although this only achieved statistical significance in volunteers treated with dutasteride (Supplemental Table 1). Serum DHT decreased with both treatments as anticipated, although this decrease was statistically significant only with finasteride.

## Discussion

In this study, dutasteride treatment was associated with hepatic insulin resistance, hepatic lipid accumulation, and decreased adipose lipid mobilization without impacting peripheral insulin sensitivity. Interestingly, both dutasteride and finasteride blunted the response of the serum metabolome to insulin. In the absence of insulin, dutasteride had a greater effect than finasteride on the serum metabolome, notably with regard to lipid intermediates (accepting that circulating cholesterol, triglycerides, and glycerol did not change). Overall, this may point to a significant contribution of SRD5A1 to metabolic homeostasis. Both 5α-reductase isoforms are expressed in the liver (7), whereas only SRD5A1 is expressed within adipocytes (8). It is likely that both isoforms contribute to GC clearance; however, the additional impact of inhibition of SRD5A1 with dutasteride may explain the more marked effects that we observed on GC metabolites and suppression of total GC metabolite production. 5α-Reductase is critical for GC clearance and androgen generation, and it remains plausible that the observations in this study may either reflect alterations in androgen and/or GC metabolism.

In vitro human and rodent models have demonstrated that GCs in isolation suppress hepatic and adipose lipogenesis. However, alongside insulin, they enhance insulin-stimulated lipogenesis (19, 25, 26). Consequentially, interventions increasing GC exposure, including 5α-reductase inhibition, may increase adipose and hepatic lipid accumulation in the presence of insulin. The additional impact of SRD5A1 inhibition to limit GC clearance with dutasteride may explain its more marked metabolic effects. The effects of androgens on lipogenesis in liver and adipose have not previously been described in detail. T limits lipid synthesis in rodent hepatocytes (27) and hypogonadism in men is associated with NAFLD (2, 3). The metabolic fate of T may also be significant because exogenously administered T funneled toward aromatization by inhibiting activation to DHT is associated with improved insulin sensitivity (28).

The findings of this study are supported by observations in rodent models. There are important distinctions when extrapolating data from rodents into the clinical setting. Importantly, rodent livers express only SRD5A1 and not SRD5A2. SRD5A1 knockout mice develop hepatic steatosis when fed an obesogenic diet, whereas SRD5A2 knockout mice do not (14, 15). Furthermore, in a model of hepatic fibrosis, SRD5A1 knockout animals developed more severe fibrotic liver disease (15). Although the observations in this clinical study may not be as dramatic as seen in rodent models, this may not only relate to differing patterns of SRD5A1 and SRD5A2 expression, but in our study, there was still some residual 5α-reductase activity as evidenced by the urinary steroid metabolite profiles. Indeed, it is possible that the potency of inhibition may explain some of the differences observed between finasteride and dutasteride. The doses of finasteride and dutasteride used in this study are those currently licensed in the United Kingdom, and therefore, it was believed that using higher doses in a healthy volunteer study was not warranted.

Clinical studies have highlighted the potential role for 5α-reductase in the regulation of metabolic phenotype, although there is still debate as to whether the abnormalities observed represent the cause or consequence of disease. Cross-sectional studies have demonstrated increasing 5α-reductase activity with insulin resistance (12) and increasing adiposity (10) and decreases after weight loss (29). A recent study examined the metabolic impact of selective SRD5A inhibition in humans (16). After a 3-month treatment period, they observed inhibition of insulin-mediated suppression of global lipolysis by dutasteride as well as a reduction in peripheral insulin sensitivity, which they suggested might reflect the role of SRD5A1 within skeletal muscle. In comparison with the current study, there are important differences to consider in terms of the duration of treatment, volunteer demographics (age, BMI), and methodology (adipose microdialysis, doses of insulin used in the clamp studies, MRS to quantify liver fat before and after treatment) as well as the analysis of the serum metabolome. Taken together, these studies would seem to complement each other and provide evidence as to the potential detrimental impact of the dual SRD5A1 and SRD5A2 inhibition.

It is important to consider the potential long-term consequences of the observations that we have made. Hepatic steatosis is a precursor to progression to more advanced stages of NAFLD including nonalcoholic steatohepatitis, and progression rates may be higher than previously thought (30). Whereas the changes and absolute amounts of intrahepatic lipid seen in this study were small, the duration of intervention was short and it remains to be determined whether longer interventions would continue to cause further lipid accumulation.

Our study has also highlighted potential mechanisms promoting lipid accumulation. Adipose tissue free fatty acid delivery is a major contributor to increased hepatic lipid accumulation (31). Subcutaneous adipose tissue lipolysis was decreased by dutasteride (and not finasteride), likely reflecting the absence of SRD5A2 in human adipose tissue. This is consistent with increased GC availability, augmenting the action of insulin suppressing lipolysis as we have previously shown in sc (and not omental) adipose in vitro and in vivo (19). The differential effects of GCs on sc and omental adipose tissue may explain the lack of significant impact on Ra glycerol. We were unable to assess hepatocyte function in the same way; however, if similar mechanisms were to operate (as has been shown in vitro), this would lead to increased hepatic lipid accumulation (26). It is likely therefore that the increase in hepatic steatosis is fueled by increased DNL, which may represent an important driver to lipid accumulation in specific subgroups of patients with NAFLD and might be more relevant than circulating NEFAs (32).

This effect of dutasteride modulating lipid metabolism is supported by the changes we saw in the serum metabolome if not as obvious in the biochemical parameters (eg, triglyceride, NEFAs, high-density lipoprotein, and low-density lipoprotein). Within the metabolome, dutasteride treatment was associated with an increase in free fatty acids as well as increased diacylglycerol and triacylglyercol and a reduction in oxidized fatty acids (as measured by the change in hydroxy fatty acids, which are intermediates in the β-oxidation pathway), suggesting that cellular metabolism is shifted away from fatty acid oxidation toward esterification pathways. The most striking finding from the fasted serum metabolome was the profound effect dutasteride treatment had on the lipid classes and in particular the glycerophospholipid (GPL) class. The finding of dutasteride increasing the GPL metabolites in tandem with the observed increased in MRS hepatic lipid content complements previously published findings of plasma and liver GPL being the greatest lipidomic discriminator of liver disease stage (33).

There are limitations of the present study. The treatment intervention was short, and despite randomization, participants randomized to finasteride had a higher BMI and higher baseline levels of intrahepatic lipid, although in other aspects, the groups were very similar. Importantly, each subject served as their own control in a paired analysis. However, we cannot exclude the possibility that baseline metabolic characteristics might impact on the potential response to these drugs. Additionally, there were only six participants within each arm of the stud, although sample size estimates had been calculated (see *Patients and Methods*). Whereas the data obtained are robust with regard to the impact on hepatic insulin sensitivity, lipid accumulation and adipose tissue function, the study was only a short-term intervention and was of insufficient duration to determine changes in body composition. The potential adverse effects in this healthy volunteer study were felt not to warrant longer-term administration, but the detailed effects upon body composition need to be systematically examined.

In conclusion, dual inhibition of 5α-reductase with dutasteride appears to convey an adverse metabolic phenotype. 5α-Reductase inhibitors are widely prescribed, and the decision to use either finasteride or dutasteride does not discriminate on the potential metabolic effects. An increased incidence of heart failure in patients treated with dutasteride has been reported (34), although data remain conflicting (35), and there is no doubt that further studies are warranted to determine the long-term metabolic consequence of 5α-reductase inhibition.
